# Editorial: Role of early life nutrition in immunomodulation and microbiota development

**DOI:** 10.3389/fnut.2023.1266725

**Published:** 2023-10-16

**Authors:** Diego G. Peroni, Cristina Campoy, Elvira Verduci

**Affiliations:** ^1^Department of Clinical and Experimental Medicine, Section of Pediatrics, University of Pisa, Pisa, Italy; ^2^Department of Pediatrics, School of Medicine, University of Granada, Granada, Spain; ^3^EURISTIKOS Excellence Centre for Pediatric Research, Biomedical Research Centre, University of Granada, Granada, Spain; ^4^Spanish Network of Biomedical Research in Epidemiology and Public Health (CIBERESP), Granada's Node, Institute of Health Carlos III, Madrid, Spain; ^5^Pediatric Department, “Vittore Buzzi” Children's Hospital, University of Milan, Milan, Italy

**Keywords:** non-communicable diseases, diet, microbiota, immune system, immunomodulation, newborn

Evidence indicates that three major factors interplay very early in life to define the healthy status of the infant: the diet, the microbiota and the immune system. Each of them is able to influence and shape the others, leading to dynamic correlations. Immunomodulation, by definition, is the modulation (regulatory adjustment) of the immune system development and function; this could be achieved through dietary intervention and the clinical impact of gut microbes and their metabolites. In fact, the neonatal and infant gut microbiome appear to be involved in gut tolerance modulation and immune system “education” (1). Breast milk contributes to the development of healthy gut microbiome containing essential micronutrients and prebiotic compounds, which support the colonization and growth of commensal bacteria, and several immune active factors, oligosaccharides and microbes. The impact of the breast feeding on the cross-talking process is relevant, involving both the microbiota and the immune system (2). Furthermore, the nourishment has a crucial role for the development of gut microbiota from the very early to late phases of life, influencing both the innate and the adaptive immune system responses (3–5). Therefore, the gut microbiome-immune system axis can be defined as a milestone in affecting the health trajectories from birth throughout adulthood. This dynamic and bidirectional cross-talking starts very early and it is particularly modulated during the first 1,000 days of life and represents an extraordinary opportunity to further investigate the maternal-offspring cross-talk the maternal education on offspring's immunity (6). The maternal nutrition, microbiota development and immunomodulation all together shape the immune responses already during pregnancy, in the womb, and then, after the birth, throughout life. These have great consequences for the prevention or onset of chronic diseases, which start during childhood and are maintained to adulthood. This is the case for the so called non-communicable diseases (NCDs), that are represented by metabolic disorders, diabetes, asthma and allergic diseases, autism and chronic inflammatory intestinal disorders. All these diseases seem to be dramatically increased in the last decades, leading to the considering of a role of both altered microbiota (dysbiosis) and malnutrition, that in turn can affect the immune responses and develop chronic diseases (3–5). Also frequent antibiotic courses during childhood could lead to persistent dysbiosis and contribute to trigger chronic diseases in adulthood (7).

Given that microbiota and immune system dialogue reciprocally, and being nutrition the basis for their development and maintenance, aim of this Research Topic was to provide and discuss evidence for a role of nutrition in shaping the microbiota and related immunomodulation. Both dietary macro- and micro-nutrients (vitamins, oligo-elements) are recognize to play a pivotal role in these mechanisms (8). Firstly, protein deficiency can lead to impaired gastrointestinal barrier function, loss of lymphoid tissue and altered intestinal microbiota (9). Also, a specific group of non-digestible carbohydrates, known as dietary prebiotics, modulate gut microbiota composition and consequently immune system. Although mechanisms are still under investigation, promising results have been shown (10). Vitamins are multifunctional compounds, belonging to the category of micronutrients, that are endowed with essential biological activities for carrying out enzymatic processes and for the health of the human organism. Some of them can exert modulating effects on the functions of the immune system, such as vitamin A, E, and D, which are implicated in the modulation of the complex molecular pathways involved in the immune response (11). Particularly micronutrient deficiencies such as iron deficiency may impact immune activation: prolonged deficit not only of iron but also of zinc, can be associated with a polarization of the immune responses of prevalent type 2 (Peroni et al.). A novel area of study is the possibility that the deficiency of these micronutrients could contribute to the etiology of the atopic diseases by affecting also the development of the microbiota (Peroni et al.). Therefore, not only over-nutrition (western diet) but also nutritional deficiencies (of macro- and micro-nutrients) can cause the dysbiosis and immunological imbalance that promote chronic diseases.

Since prevention strategies represent a fascinating challenge for every researcher, this was discussed in a mini-review (Coppola et al.), that considered the potential role of preventive and therapeutic immune-nutrition strategies applied to pediatric food allergy. Food allergy is a model to study the cross-talk among gut microbiota and the immune system. Strategies for modulating the microbiota during childhood have been discussed: an early and integrated approach could prevent and counteract gut dysbiosis and its effects. By modulating the interplay among dietary factors, gut microbiota and immune system, it would be possible to consider the concept of “immune-nutrition”, that is actually emerging as a novel frontier (12). This has been clearly reviewed (Coppola et al.) considering that if the immune-nutrition approach would be applied early in life, this could represent a new strategy to prevent and to potentially treat the non-communicable diseases. In the specific case of food allergy, an integrated proactive approach could be able to speed up the oral tolerance acquisition though the influence on the gut microbiota-immune system axis.

A study on animal model published in the Research Topic, demonstrated that the maternal *Lactobacillus rhamnosus* administration was able to impact on neonatal CD4 T-cell activation. Interestingly, the result of *Lactobacillus rhamnosus* and immune system crosstalk prevented also the development of a T helper 2-type allergic airways disease (Smout et al.). These results lead to consider that also probiotic administration, probably in special window of opportunity, could interact with the gut microbiota and the immune-system (13).

Another paper from the Research Topic describes the rationale of a prospective birth cohort study investigating the link among early life malnutrition, the microbiota and the health outcomes (Tamarelle et al.). The VITERBI GUT project will enroll under-nourished, normal-nourished and over-nourished mothers at the third trimester of pregnancy and the offsprings until 2 years of life. The microbiota composition of maternal and child fecal and oral samples as well as breast milk and vaginal samples, will be determined in order to characterize the microbiota though these trajectories, where the nutrition could shape the microbiota and be related to the onset of metabolic disease later in life. Finally, another paper considered several environmental factors that could act as contaminants in early life nutrition. This is the case for the endocrine-disrupting chemicals which could affect the newborn immune system and the gut microbiota, leading in turn to modifications of the metabolism (Street et al.). Exposure to these substances may induce all pro-inflammatory effects such as tissue inflammation, lymphocytes polarization, increase of pro-inflammatory cytokines.

In summary, there is great interest to study the connections among the diet, the microbiota, and the immune system. The concept of immune modulation is emerging and leads to fascinating prospective in order to prevent or treat, if already established, several disorders all comprised in the category of the non-communicable diseases. All this evidence highlights the importance of a balanced diet from the pregnancy, early life, thought adulthood to minimize and/or prevent health problems.

## Author contributions

DP: Writing—original draft. CC: Writing—review and editing. EV: Writing—review and editing.
